# Effect of Labelling and Information on Consumer Perception of Foods Presented as 3D Printed

**DOI:** 10.3390/foods11060809

**Published:** 2022-03-11

**Authors:** Xiaoqin Feng, Khemiga Khemacheevakul, Susana De León Siller, John Wolodko, Wendy Wismer

**Affiliations:** Department of Agricultural, Food and Nutritional Science, 4-10 Agriculture Forestry Centre, University of Alberta, Edmonton, AB T6G 2P5, Canada; xiaoqin3@ualberta.ca (X.F.); khemache@ualberta.ca (K.K.); deleonsi@ualberta.ca (S.D.L.S.); jwolodko@ualberta.ca (J.W.)

**Keywords:** 3D food printing, novel food technology, food technology neophobia, acceptance, preference

## Abstract

Labelling and information have been shown to increase acceptance of novel food technologies. The novel technology of 3 Dimensional Printing (3DP) of foods is not well known among consumers. The study aim was to investigate the effect of the 3DP label and benefits information on consumer acceptance and perception of plausible 3DP foods. Commercially available foods, such as milk chocolate swirls, gummy candy carrots, and baked potato Smiles^®^, represented 3DP benefits, and each was evaluated in a sensory panel. Participants rated acceptance and perceived quality after each of three product presentations; first labeled “conventional”, then labeled “3D printed”, and again labeled 3D printed after information presentation. Participants indicated product preference after the third presentation. Food Technology Neophobia (FTN), attitude, and previous 3DP knowledge were queried. Quality rating of chocolate swirls and gummy candy carrots increased when labeled as 3DP versus conventional; information did not further increase quality ratings. Participants preferred 3DP chocolate swirls and gummy candy carrots to conventional in the final evaluation. Label and information did not change flavor, texture, or overall acceptance ratings for any product. Attitude towards 3DP of foods increased with lower FTN. Future studies could tailor information to consumer interests and knowledge gaps that highlight relevant benefits of 3DP.

## 1. Introduction

Three-dimensional (3D) printing is a production technology that allows successive deposition of materials layer by layer based on computer-aided design [1]. Three-dimensional food printing (3DFP) applications include creative design of confections [2] through customization of intricate food geometries [3], personalized nutrient delivery [4,5], appealing presentation of puréed diets for individuals with chewing and swallowing challenges [6,7], valorization of food waste [8], and meat substitute fabrication [9]. Considering these benefits, there is great optimism about the future of this novel food technology [2,10]. Recent 3DFP research has emphasized technological advancement and product prototyping or development [11]. Consumer acceptance of this novel technology and its resulting products is an important, under-investigated determinant of the future success of 3DFP to realize the aforementioned benefits [12,13].

Initial consumer acceptance of 3DFP products was based on the evaluation of the 3DFP concept or pictures of 3D printed foods [12,13,14] with few tasting experiences of the products. When actual 3DP products have been compared to their conventional counterparts, military personnel preferred the sensory attributes of customized 3DP bars compared to the original conventionally produced formulation [15] and the appearance acceptance of chocolate was influenced by the infill level of the honeycomb pattern [16].

Several recent publications have indicated a positive effect of food product labelling and presentation of positive information on consumer sensory acceptance of novel foods and food technologies. Labels indicating quality, content, and production method increased consumer sensory acceptance or perceptions of sustainable chocolate, salt-reduced potato chips, and pasteurized over Ultra High Temperature (UHT) processed milk [17,18,19]. The influence of the 3DP label on product sensory acceptance relative to conventionally produced foods has been performed previously using a variety of approaches. Manstan et al. [20] compared sugar cookies labeled as 3D printed and conventionally produced; both cookies were made from a single cookie dough formulation. The manipulated appearance of the product labeled 3DP resulted in higher appearance acceptance ratings with no other differences in overall and sensory attribute ratings.

An approach to evaluate the effect of labelling and information on consumer sensory acceptance of foods produced by novel technologies is to change the label and information at successive presentations of a single product. After receipt of positive information about cultured meat, participants in the study by Rolland et al. [21] rated the taste of conventionally made hamburgers labeled as “cultured” to be better than the identical product labeled as “conventional”. In contrast, labels of “not nanotechnology produced” or “nanotechnology produced” with information about the benefits of the technology did not affect sensory attribute acceptance of cherry tomatoes and chocolate ice-cream presented under both label conditions [22] and labelling young boar and pork samples as either meat type did not affect consumer sensory acceptance of the meat [23]. This approach has not been trialed in studies of with 3DP foods.

In addition to product sensory acceptance, consumer attitude towards 3DFP will also determine its success. Consumer skepticism of novel food technologies limits their widespread application [12,24]. Reasons for skepticism of novel food technologies and novel foods include a lack of knowledge about the technology [25,26] and a lack of perceived benefits of the technology [27,28], food neophobia [29], and food technology neophobia (FTN), described as consumer reluctance to accept foods produced by new technologies [30]. Providing positive information about novel foods or related food technologies has been identified as a strategy to improve consumer attitude through increased knowledge [31].

Consumer knowledge, FTN, benefit perceptions, and the effect of positive information on consumer attitude have been previously evaluated in the context of 3DFP. Consumer knowledge about 3D printed foods was low in a Swiss sample [12] and an Atlantic Canadian sample [13]; however, two-thirds of Australian university participants had high self-assessed knowledge about 3D printing and 3DFP, and around half showed a clear understanding of 3DFP, suggesting a high knowledge level in the young and highly educated population [16]. A lower FTN score predicted a higher consumer initial attitude towards 3D printed foods and attitude after presentation of positive information [12], and a low FTN in a Dutch military environment indicated a food technology neophilic population [15]. While consumer participants in some studies have identified 3D printed foods as healthy [13] and acknowledged benefits of 3DFP [16], others have described 3D printed foods as unfamiliar, unnatural, unhealthy, and non-food like [14]. Brunner et al. [12] observed an increased positive consumer attitude towards 3DFP after receipt of positive information about 3DFP, although overall attitude ratings after information remained negative. Caulier et al. [15] did not observe a difference in attitude of Dutch soldiers before and after presenting information about 3DFP and its benefit to personalized nutrition in the military setting; however, attitude ratings increased after repeated tastings of 3D printed foods. Additionally, Brunner et al. [12] observed that consumer orientation to convenience predicted a positive initial attitude and attitude change towards 3D printed food after receipt of positive information about 3DFP.

The primary aim of the study was to investigate the effect of labelling and product-specific positive information about 3DFP on consumer sensory acceptance of foods labeled as 3D printed relative to their initial presentation as conventionally produced, incorporating product taste evaluations with each assessment. This approach to the evaluation of 3DFP food products has not been previously explored. Secondary objectives were to determine the impact of labeling and information on consumer attitude towards ‘3D printed foods’, and the effect of FTN and previous knowledge about 3D printing on acceptance, perceived quality, and attitude towards 3D printed foods. Consumer orientations to health, natural content, convenience, and familiarity with digital technology and comments on tasted products were also collected.

## 2. Materials and Methods

### 2.1. Study Design

Three food products were selected for this study, each representing an established benefit of 3DFP. Milk chocolate swirls (Carnaby Sweet, Toronto, ON, Canada) represented the 3DFP benefit of creative, visually pleasing product design. Gummy candy carrots (Bulk Barn Foods Ltd., Aurora, ON, Canada) represented personalized nutrition in gummy vitamin supplement form, a popular presentation in North America for both adults and children. The appealing presentation of pureéd foods was demonstrated through baked potato Smiles^®^ (McCain Foods Ltd., Florenceville, NB, Canada), a formed product described by the manufacturer as ‘grin-shaped mashed potato bites’.

The study design used to evaluate the effect of labeling and information was guided by other authors [21,23] who presented a single product under differing labelling and information conditions, i.e., holding the product constant while varying the presentation label. Study food products were purchased from local grocery stores. These food products have limited availability and would have been unfamiliar to many participants, and their unique product shapes lent credibility to their presentation as 3D printed foods. Each food product was evaluated in its own sensory panel.

**Figure 1:**
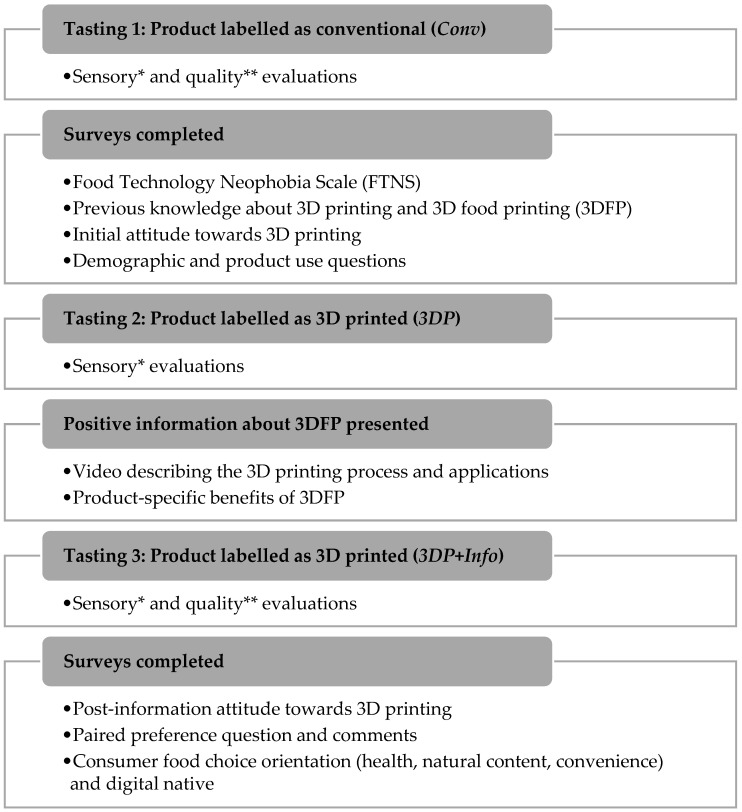
Study design for the assessment of study food products and survey completion. * Sensory evaluations: Overall liking and liking of appearance, aroma, flavor, texture on 9-point hedonic scales anchored with ‘dislike extremely” to “like extremely”. ** Quality evaluations: Agreement with high product quality on 5-point Likert scales anchored with “strongly disagree” to “strongly agree”.

In each panel, participants were seated in individual sensory booths under natural white lighting and completed all assessments on Compusense Cloud software using a tablet (Figure 1). Participants tasted and evaluated acceptance and quality over three monadic presentations of a single food, presented first as conventional (*Conv*), then 3D printed (*3DP*), and 3D printed again after the presentation of information and product-specific benefits about 3DFP (*3DP + Info*) (Table S1). The information presentation consisted of a short video [32] to overview the process of 3D printing, followed by product-specific text and images to describe the benefits of 3DFP associated with the food product.

After sensory evaluations were completed, participants indicated their preference between samples labeled as conventional and 3D printed. Between product evaluations, participants completed surveys of attitude towards 3D printing, orientations to *health, natural content, convenience*, and *familiarity with digital technology*, the Food Technology Neophobia Scale (FTNS), and indicated their previous knowledge about and attitude towards 3D printing (Figure 1).

### 2.2. Participants

Participants who indicated a willingness to sample 3D printed foods and regular consumption of the general category of the study product (i.e., special occasion chocolates for chocolate swirls [n = 68], vitamin supplements for gummy candy carrots [n = 59], puréed foods for potato Smiles^®^ [n = 59]) were recruited from the University of Alberta community (Edmonton, AB) and invited to participate in one consumer sensory panel. Participants completed written informed consent and received a $5 gift card at the end of the study. The study protocol was approved by a Research Ethics Board at the University of Alberta (Pro00089544). As institutional ethical guidelines consider the presentation of a product with a false label to be a deceptive practice, after all data collection participants were debriefed via email about the true identity of the study samples (i.e., none were 3D printed) and could withdraw their data from the study within one week.

### 2.3. Food Sample Preparation

Chocolate swirls and gummy candy carrots were stored in air-tight containers at room temperature, and potato Smiles^®^ were stored in a freezer (−18 °C) until preparation. Chocolate swirls and gummy candy carrots were served directly in 30mL plastic cups with lids at room temperature, one piece per container. Potato Smiles^®^ were baked as per manufacturer instructions. One prepared potato Smiles^®^ was served in a lidded 237 mL Styrofoam cup. Samples were kept warm until serving. Misshapen samples of any product were discarded.

### 2.4. Product Assessments and Surveys

#### 2.4.1. Product Assessments

Sensory evaluation was performed after each product tasting; overall acceptance and acceptance of appearance, aroma, flavor, and texture of food samples were evaluated on 9-point hedonic scales anchored from “dislike extremely” to “like extremely”. Participants were invited to provide comments about the food sample. Agreement of high product quality was evaluated on 5-point Likert scales anchored from “strongly disagree” to “strongly agree”.

After the last tasting, participants completed a paired preference of samples labeled as “conventional” and “3D printed” and were invited to provide comments about their preference.

#### 2.4.2. Surveys

Constructs known to influence the acceptance of novel food technologies guided the selection of questions included in the research [12]. Questions to assess previous knowledge and attitude towards 3D printing were adapted from Brunner et al. [12]. Self-assessed previous knowledge about 3D printing and 3DFP were evaluated using 5-point category scales anchored from “not at all” to “extremely”. Attitude towards 3D printing before and after tastings and receipt of positive information about 3DFP were evaluated on 7-point semantic differential scales anchored from “negative” to “positive”.

Food Technology Neophobia was quantified on the FTNS [30]. Participants rated their agreement to 13 items about new food technologies on 7-point Likert scales from “totally disagree” to “totally agree”.

Consumer food choice orientations to *health*, *natural content*, and *convenience*, and familiarity with digital technology (*digital native*) were assessed using the question items adapted by Brunner et al. [12] for their study of 3DFP, originally developed and validated for previous studies [33,34,35]. Participants rated their agreement to each item on 7-point Likert scales from “strongly disagree” to “strongly agree”.

Participant age, education, income level, and household size data were collected. Participants identified their frequency of consumption of the general category of the study product; special occasion chocolate (chocolate swirl panel), nutritional supplements (gummy candy carrot panel), and familiarity with pureéd diets (potato Smiles^®^ panel; http://www.mccainpotatoes.com/products/smiles (accessed on 21 February 2022)).

### 2.5. Data Analysis

Data were analyzed using R statistical language (R Core Team, 2020) SensoMineR and Compusense Cloud sensory software (Compusense, Guelph, ON, Canada). A significance level of *p* ≤ 0.05 was used in all statistical tests. Within-Subjects Analysis of Variance (ANOVA) and pairwise *t*-test with Bonferroni correction were used to determine differences in sensory acceptance scores and perceived quality among the three consecutive tastings, and a paired two sample *t*-test was used to compare initial and final attitude scores towards 3DFP.

One-way ANOVA indicated no difference in FTN scores among participants in the three product panels. Subsequently, data from all three product panels were merged and then stratified by FTN median score to create food technology neophilic and less neophilic groups. Previous knowledge about 3D printing was greater among the potato Smiles participants (*p* < 0.01). However, as participants of the three panels were a homogeneous university population, merged data were also stratified as knowledgeable and not knowledgeable by collapsing categories on the scale to assess previous knowledge about 3D printing. Overall acceptance, perceived quality, and attitude scores across all products were compared using Tukey’s HSD where appropriate and between stratified groups of each variable using an unpaired two sample t-test.

Demographic and product use information, consumer orientations, and change in acceptance ratings between two adjacent sample presentations of each food product were analyzed using descriptive statistics.

Participant comments about the food products were analyzed by content analysis [36] by three authors (XF, KK, SS). Only word categories with a frequency of 5% and 10% of all citations were included in the presentation of preference and sensory acceptance comments, respectively.

## 3. Results

### 3.1. Participant Characteristics

A total of 186 individuals participated in the chocolate swirl (n = 68), gummy candy carrot (n = 59), and potato Smiles^®^ (n = 59) panels. The majority of participants (53–75%) in all panels were between the age of 18–25 years (Table 1), and nearly all (93–96%) had some or completed university or higher level of education, reflecting recruitment on a university campus. Nearly half or more of participants (49–73%) were knowledgeable (“somewhat” to “extremely”) about 3D printing at the start of the panel, while the majority (80–93%) had little knowledge (“not very” or “not at all”) of 3DFP. The average FTN score (43.8–45.7) was lower than the midpoint of the FTNS (52) indicating a tendency towards food technology neophilia. Participants were highly oriented to health, natural content, and convenience in their food choice and were digital natives. Half of the participants (50%) in the chocolate swirl panel frequently consumed special occasion chocolates and roughly half (54%) in the gummy candy carrot panel frequently consumed nutritional supplements. The majority of participants (66%) in the potato Smiles^®^ panel were familiar with pureéd diets.

### 3.2. The Effects of Label and Information on Sensory Attribute Acceptance and Quality

Over the three product presentations, sensory attribute and overall acceptance of chocolate swirls, gummy candy carrots, and potato Smiles^®^ were rated as “liked slightly” to “moderately” (Table 2). There were no significant differences in acceptance scores of flavor, texture, and overall acceptance of the three food products over the three labelling and information presentations. Appearance liking of chocolate swirls was not different between the *3DP* and *3DP + Info* presentations; both were rated higher than the *Conv* counterparts. The aroma of gummy candy carrots was liked more when samples were presented as *3DP + Info* compared to *3DP*. The aroma of potato Smiles^®^ was liked more when samples were presented as *Conv* compared to *3DP + Info*, but liking for either presentation was not different from liking for the *3DFP* label.

Participant comments about the sensory attributes of food samples labeled as *Conv* and *3DP* were grouped around the five dimensions of texture, taste/flavor, appearance, quality, and similar/same (Table 3). The samples labeled as *3DP + Info* elicited very few participant comments. Texture, taste/flavor, and the two samples being similar/same were the most frequently mentioned dimensions. *Smooth mouthfeel* was mentioned more frequently when the chocolate swirls were labeled as *3DP* as compared to *Conv,* while negative textural perceptions including *greasy/waxy* and *dry/grainy* were no longer mentioned. Chocolate swirls labeled as *Conv* were described as *too sweet*, *positive, milky*, and of *average* and *low quality,* while the same product labeled as *3DP* had *rich chocolate flavor* and was *tasty,* but was *bland* for some. The texture of the *Conv* gummy candy carrots was described as *chewy/hard*; when labeled as *3DP* it was perceived to be *less chewy*. The taste/flavor of gummy candy carrots was perceived to be *stronger* by some participants when labeled as *3DP*, and *appealing* and having *good taste/flavor* in both presentations. The dominant texture description of *Conv* potato Smiles^®^ was *not crispy*, which was less frequently mentioned when presented as *3DP*. A *good/less mushy* texture and *tasty* were only mentioned when the potato Smiles^®^ were labeled as *3DP*.

For all three products, participants’ mean ratings were “neither agree nor disagree” or “agree” that the product was of high quality when presented as *Conv* (Table 2). A significant increase in quality perception when presented as *3DP* and *3DP + Info* compared to *Conv* was observed for the chocolate swirls and gummy candy carrots.

### 3.3. The Effect of FTN and Previous Knowledge about 3D Printing on Overall Opinion, Perceived Quality and Attitude towards 3DP

Compared to the less FT neophilic group, the FT neophilic group gave higher ratings of overall opinion and perceived quality for products presented as *3DP* and *3DP + Info* compared to *Conv* (Table 4). The overall opinion ratings of the less FT neophilic group did not change over the three product presentations. There were no significant differences in overall opinion and perceived quality between participants who were knowledgeable and not knowledgeable about 3D printing.

For each of the three study products, participant initial attitude towards 3D printing was positive (mean = 5.2; SD = 1.0–1.2), and was more positive (mean = 5.9–6.0; SD = 0.9–1.0) (*p* < 0.001) at the conclusion of the study. Both FT neophilic and less FT neophilic groups had a positive initial attitude; however, the FT neophilic group had a higher attitude score compared to the less neophilic group at both timepoints (Table 4). There was no significant difference in attitude between groups that were knowledgeable and not knowledgeable about 3D printing. Attitude became more positive for both groups from the first to the second evaluation.

### 3.4. Paired Preference of Samples Labeled as Conventional and 3D Printed

In the paired preference test, chocolate swirls and gummy candy carrots labeled as 3D printed were preferred to their *Conv* counterparts (75% vs. 25% and 79% vs. 21%, respectively) while there was no preference for potato Smiles^®^ (59% vs. 41%). The majority of participants (92% and 97%) in chocolate swirl and potato Smiles^®^ panels described perceived product differences motivating their preference (Table 5). A *good/better* sensory profile was most frequently mentioned when explaining preference for samples of either label. For both products, some participants who preferred the “3D printed” samples mentioned their support for technology that is *interesting* and *novel* and perceived benefits of 3DFP in fabricating *creative, custom, appealing design*. Perceived benefits of 3DFP specific to chocolate swirls were *cost effective* food production and *more efficient production*. The *Conv* sample was preferred by participants who cited perceived benefits, knowledge and familiarity of the conventional product, and lack of visual appeal of the 3D printed product. The *Conv* potato Smiles^®^ was described as *more natural/healthy*. Some participants who preferred the *Conv* chocolate swirls mentioned that the *3D design was not cool enough* and that they had a *lack of knowledge about 3DFP* or were *more knowledge about the conventional product*. About 25% or fewer of the participants identified no difference (*similar/same* or *no preference*) between the food samples presented as *Conv* and 3D printed. Some participants who preferred the *Conv* potato Smiles^®^ indicated an accepting attitude towards 3DFP in the future.

## 4. Discussion

Our study objective was to determine the influence of label and information about 3D food printing on perceived product quality, attitude towards 3DFP and 3DP, and sensory acceptance of three food products relative to their initial presentation as conventionally produced. For two of the three study products, the 3DP label, but not information, influenced the perception of the product. The 3DP label increased the perception of product quality but did not increase sensory attribute acceptance ratings; however, participants described sensory attributes and quality of products with the 3DP label more positively than their conventional counterparts. Use of a single conventionally produced product presented under three labelling conditions permitted an explicit comparison of consumer perception of the impact of labelling and information, an approach not used previously with ‘3DFP’.

The 3DP label increased quality ratings of chocolate swirls and gummy candy carrots over the same product labeled as conventionally produced, and participant comments suggested enhanced product quality attributes were perceived, such as ‘better’ taste, texture, and flavor. In contrast, acceptance ratings of product sensory attributes and overall product acceptance, in general, did not increase with the presence of the 3DP label. Some participants correctly perceived the products to be the same, while other participants commented that they perceived specific sensory attribute differences between the products labeled as 3DP and conventional. A greater number of positive, and fewer negative, sensory attributes comments were received for the 3DP labeled product compared to the product labeled as conventional. The clear paired preference selection of 3DP labeled chocolate swirls and gummy candy carrots at the final evaluation reflects the greater perceived quality of these products over their conventional counterparts. This difference may be because paired preference, a form of hedonic ranking, discriminates among samples while hedonic ratings may not when hedonic differences between products are small [37].

Previous studies of 3DP versus conventionally labeled products also demonstrate a positive influence of the 3DP label on quality perception but not sensory acceptance, and a lack of influence of information about 3DFP on product perception. Manstan and McSweeny [13] evaluated the effect of labelling on the perceptions of 3DFP vs conventional foods using pictures of these foods. While the attribute ‘quality’ was not specifically assessed, study participants in general perceived the 3DFP products to be healthier than their conventional counterparts.

In the literature, sensory attribute ratings of 3DP foods differ from their conventional counterparts only when a true difference exists between products. A measurable greater complexity of visual design in the 3D printed product resulted in increased sensory acceptance appearance ratings for both 3DFP chocolates and sugar cookies, but did not influence overall acceptance or flavor and texture attribute acceptance ratings [16,20]. Consumer evaluations of 3DP foods’ sensory attributes are useful to provide insight about acceptance of food formulations developed specifically for 3DP foods [8,38]. In contrast to some other food production labels such as ‘organic’, in which the label confers superior sensory attributes in the final food product compared to conventionally produced counterparts [39], our results and others indicate that sensory acceptance is not influenced by the 3DP label unless attribute differences are apparent.

The presentation of information about the 3D food printing process and its benefits did not increase sensory attribute acceptance or perceived quality for any product, with the exception of the aroma of gummy candy carrots. Previous studies of novel technologies suggest that information must be tailored to the needs of the consumer to generate positive attitude change [40]. While our study participants indicated they consumed food products similar to the study food they evaluated, the information presented may not have resonated with their needs and interests, i.e., potato Smile^®^ panel participants were familiar with pureéd foods, they did not consume them. Health care professionals and individuals on pureéd food diets would likely have greater appreciation of this application of 3DFP. Similarly, personalized nutrient supplementation and visually appealing confectionary did not resonate strongly with the study’s GenZ university participants. Three-dimensional food applications targeted to this generation could instead highlight sustainability and ‘social responsibility’ applications [41] such as printed snacks from fruit and vegetable waste [5,8] and printed plant based ‘meat’ and cultured animal cells [9]. Additionally, information about a novel technology may be beneficial to consumers when the technology is unfamiliar or where misconceptions are common, such as food irradiation [42]. Beghin and Gustafson [43] identify that plant-engineering techniques that provide benefits to foods not available in conventional products leads to higher consumer valuation of the novel products. Thus, information tailored to targeted consumer interests and knowledge gaps that highlights benefits of 3DP over conventional product will be of greatest relevance to consumers.

The second study objective was to determine the impact of labeling and information on consumer attitude towards 3D printed foods, and the effect of FTN and previous knowledge about 3D printing on acceptance, perceived quality, and attitude towards 3D printed foods. Compared to the initial assessment, participant attitude towards 3DP was more positive after receipt of information and the opportunity to taste the products. This was also observed by Caulier et al. [15] and by Manstan et al. [20], who suggested that the positive tasting experience with the study food product presented as 3DP generated greater acceptance of 3D food products in general.

As anticipated, food technology neophilia was associated with a more positive perception of 3DP among our university-based participants [12,15]. Greater acceptance of novel technologies is characteristic of younger consumers [44,45]. As FTN decreases consumer overall acceptance of 3DP products, it would be valuable to identify the specific attributes of this novel technology that reduce or inhibit its acceptance. Previous studies with consumers on the topic of 3DFP have identified concerns of poor taste, and health and safety risks [46] and novel food production technologies and their products are viewed as unnatural by some consumers [47]. The presentation of information that addresses these concerns may result in greater acceptance of 3DFP.

Previous knowledge about 3DP did not associate with product acceptance, perceived quality or attitude towards 3DP. Brunner et al. [12] also observed that initial knowledge level of 3D printed foods did not predict consumer attitude before and after receipt of positive information about 3DFP; and that higher initial knowledge level resulted in smaller attitude change after receiving positive information. High consumer initial knowledge could indicate high confidence in an existing attitude and high resistance towards attitude change [31].

### 4.1. Future Studies

In their review, Siegrist and Hartmann [47] identify factors that determine consumer acceptance of new technologies which could be applied in future studies of 3DFP acceptance. Consumer opinions of novel technologies are changeable [47]; thus, further research to understand consumer barriers to acceptance of 3D printing technology and foods generated from it may result in greater acceptance when these barriers are identified and addressed. Future studies could explore ‘psychological characteristics’ of consumers shown to influence acceptance of novel technologies and novel foods. Lin et al. [48] associated the personality trait of ‘openness’ to consumer acceptance of GM pork. Variety seeking in the diet [49] and curiosity [50] have both been identified as motivators of insect-based food consumption, and self-described ‘adventurous eaters’ indicated a willingness to try 3DP foods [46].

In this study, health, natural content, convenience, familiarity with digital technology, and consumption frequency of the study products were analyzed as descriptive variables. Inferential statistical analysis of the relationship between those constructs and acceptance of tasted 3D printed foods could be performed in future studies. Additionally, as FTN decreases consumer acceptance of 3D printed foods, future research could investigate psychosocial determinants of FTN and interventions for its reduction in the context of 3DFP. Comprehensive tools rather than single questions could be used to assess knowledge and attitude towards 3DP technology [12,13,16] among more demographically diverse participants.

### 4.2. Study Limitations

Three-dimensional printed food designs are typically more visually complex than the products presented in this study; thus, participants may have expected products with intricate geometries characteristic of this technology. The warming method for the potato Smiles^®^ resulted in variable product quality and a lack of impact of labelling that was observed for the other two study products.

Our study participants were drawn from a university and not the general population; the COVID-19 pandemic prevented completion of this study with off-campus participants who would have represented a larger and more diverse participant base. The university community participants, however, are representative of the young and educated population who show greater acceptance towards new technologies [13,22,51] and are likely to be the early adopters of 3DFP.

## 5. Conclusions

A novel aspect of this study was the use of a single conventionally produced food product presented under three labelling conditions to permit an explicit comparison of consumer perception of the impact of labelling and information. The 3DP label, but not information, increased the perception of product quality, but did not increase sensory attribute acceptance ratings for two of three study products. Participants described sensory attributes and quality of products with the 3DP label more positively than their conventionally labeled counterparts and preferred the 3DP labeled products in the paired preference evaluation. However, in contrast to food production labels such as ‘organic’, which is associated with superior sensory attributes in the final food product, sensory acceptance is not influenced by the 3DP label unless sensory attribute differences are apparent, such as visual complexity.

The presentation of information about the 3D food printing process and its benefits did not increase sensory attribute acceptance or perceived quality of the study products, however participant attitude towards 3DP was more positive after receipt of information and the opportunity to taste the products. Future studies should tailor information to target specific consumer interests and knowledge gaps that highlight relevant benefits of 3DP over conventional products.

Food technology neophilia was associated with a more positive perception of 3DP.

As consumer opinions of novel technologies are changeable, the presentation of information that addresses concerns of food technology neophobia and identification of consumer ‘psychological characteristics’ known to influence acceptance of novel technologies and novel foods may result in greater acceptance of 3DFP and its product.

## Figures and Tables

**Table 1:** Participant demographics, food technology neophobia, and attitude orientation scores for the chocolate swirl (n = 68), gummy candy carrot (n = 59), and potato Smiles^®^ (n = 59) evaluations.

	Chocolate Swirl	Gummy Candy Carrot	Potato Smiles^®^
	n (%)	n (%)	n (%)
Age (years)			
18–25	36 (53)	44 (75)	35 (59)
26–35	18 (26)	8 (14)	22 (37)
36 and older	14 (21)	7 (12)	2 (3)
Education			
Some or completed high school	3 (4)	4 (7)	3 (5)
Some or completed post-secondary	42 (62)	33 (56)	29 (49)
Some or completed postgraduate	23 (34)	22 (37)	27 (46)
Annual Income ^1^			
< $36,000	18 (26)	24 (41)	30 (51)
$36,001–$71,000	13 (19)	9 (15)	9 (15)
$71,000–$115,000	15 (22)	9 (15)	8 (14)
>$115,000	8 (12)	3 (5)	2 (3)
Prefer not to disclose	14 (21)	14 (24)	10 (17)
Household size			
1–2	34 (50)	26 (44)	31 (52)
3–4	27 (40)	21 (36)	25 (43)
≥5	7 (10)	12 (20)	3 (5)
Previous knowledge about 3D printing ^2^			
Knowledgeable	33 (49)	33 (56)	43 (73)
Previous knowledge about 3D food printing ^2^			
Knowledgeable	5 (7)	12 (20)	12 (20)
	Mean (sd)	Mean (sd)	Mean (sd)
Food technology neophobia ^3^	45.1 (10.3)	45.7 (9.9)	43.8 (9.7)
Health orientation ^4^	35.7 (3.7)	34.6 (5.5)	34.1 (5.6)
Natural content orientation ^4^	14.5 (3.9)	14.6 (4.3)	14.8 (4.3)
Convenience orientation ^4^	23.5 (7.6)	24.1 (5.2)	22.2 (7.8)
Digital native orientation ^4^	45.7 (6.0)	45.5 (5.7)	45.6 (7.8)

^1^ Categories reflect tax brackets in Canadian dollars. ^2^ “Somewhat” to “extremely” categories on a 5 point scale anchored from “not at all” to “extremely” ^3^ Agreement to 13 items about new food technologies on 7-point Likert scales from “totally disagree” to “totally agree”. ^4^ Agreement to each item on 7-point Likert scales from “strongly disagree” to “strongly agree”. **^®^** Registered trademark.

**Table 2:** Mean ^1^ sensory acceptance ^2^ and perceived quality ^3^ scores ± SD for chocolate swirls (n = 68), gummy candy carrots (n = 59), and potato Smiles^®^ (n = 59) when presented as conventionally produced (Conv), 3D printed (3DP), and 3D printed with product benefit information (3DP + Info).

	Conv	3DP	3DP + Info
Appearance			
Chocolate swirl	6.7 ± 1.5 ^a^	7.2 ± 1.3 ^b^	7.1 ± 1.2 ^b^
Gummy candy carrot	6.7 ± 1.5	6.8 ± 1.5	6.9 ± 1.4
Potato Smiles^®^	7.3 ± 1.1	7.3 ± 1.2	7.4 ± 1.2
Aroma			
Chocolate swirl	7.0 ± 1.2	7.2 ± 1.3	7.2 ± 1.1
Gummy candy carrot	5.6 ± 1.3 ^ab^	5.5 ± 1.2 ^a^	5.8 ± 1.2 ^b^
Potato Smiles^®^	7.2 ± 1.1 ^a^	6.9 ± 1.3 ^ab^	6.8 ± 1.4 ^b^
Flavor			
Chocolate swirl	7.2 ± 1.4	7.4 ± 1.3	7.3 ± 1.1
Gummy candy carrot	6.6 ± 1.5	6.8 ± 1.4	6.9 ± 1.2
Potato Smiles^®^	6.8 ± 1.3	6.7 ± 1.4	6.7 ± 1.4
Texture			
Chocolate swirl	7.2 ± 1.5	7.3 ± 1.1	7.3 ± 1.1
Gummy candy carrot	6.0 ± 1.8	6.0 ± 1.7	6.2 ± 1.7
Potato Smiles^®^	6.4 ± 1.6	6.0 ± 1.7	6.3 ± 1.7
Overall opinion			
Chocolate swirl	7.1 ± 1.3	7.4 ± 1.3	7.4 ± 1.1
Gummy candy carrot	6.5 ± 1.2	6.5 ± 1.3	6.8 ± 1.3
Potato Smiles^®^	6.8 ± 1.2	6.7 ± 1.4	6.7 ± 1.5
Perceived quality			
Chocolate swirl	3.2 ± 1.0 ^a^	3.7 ± 0.9 ^b^	3.7 ± 0.8 ^b^
Gummy candy carrot	3.2 ± 1.0 ^a^	3.6 ± 0.8 ^b^	3.7 ± 0.8 ^b^
Potato Smiles^®^	3.6 ± 0.8	3.7 ± 0.9	3.8 ± 0.9

^1^ Mean scores with different superscript letters in the same row are significantly different (*p* ≤ 0.05). ^2^ Evaluated on 9-point hedonic scales anchored from 1 = “dislike very much” and 9 = “like very much”. ^3^ Agreement of high product quality evaluated on 5-point Likert scales anchored from 1 = “strongly disagree” and 5 = “strongly agree”. **^®^** Registered trademark. ^a,b^ Different superscripted letters indicate statistical difference (*p* ≤ 0.05).

**Table 3:** Participant sensory perception comments ^1^ of identical food samples labeled as conventional and 3D printed.

Conventional Label			3D Printed Label		
Chocolate Swirl ^2^					
Dimensions	Categories	Frequency of Mention (%) ^3^	Dimensions	Categories	Frequency of Mention (%) ^3^
Texture		38	Texture		39
	Greasy/waxy	13		Smooth mouthfeel	29
	Dry/grainy	13		Good	10
	Smooth mouthfeel	13			
Taste/flavor		55	Taste/flavor		35
	Too sweet	18		Rich chocolate flavor	14
	Positive taste/flavor attributes	15		Tasty	10
	Tasty	13		Bland	10
	Milky	10			
Quality		45	Similar/same		35
	Low quality	23		Similar/same taste/flavor	18
	Average	23		Similar/same overall	16
Gummy candy carrot ^2^					
Texture		81	Texture		56
	Chewy/hard	70		Less chewy	26
	Good hardness/mouthfeel	11		Chewy/hard	21
				Better	10
Taste/flavor		19	Taste/flavor		28
	Appealing	19		Good taste/flavor	18
				Stronger	10
Appearance		11	Appearance		10
	Attractive	11		Impressed	10
			Similar/same		69
				Similar/same appearance	23
				Similar/same overall	18
				Similar/same taste/flavor	15
				Similar/same texture	13
Potato Smiles^® 2^					
Texture		72	Texture		45
	Not crispy	44		Not crispy	27
	Crispy	14		Good/less mushy	18
	Dry/grainy	14			
Taste/flavor		17	Taste/flavor		27
	Bland	17		Bland	16
				Tasty	11
			Similar/same		30
				Similar/same overall	30

^1^ Categories mentioned by least 10% of commenting participants were included for content analysis. ^2^ Participants providing comments in each panel for the conventional label and the 3D printed label, respecitley, were chocolate swirl (n = 40; n = 49); gummy candy carrot (n = 37; n = 39); and potato Smiles^®^ (n = 36; n = 44). ^3^ Each respondent could enter multiple responses therefore frequency percentages total more than 100%.

**Table 4:** Mean ^1,2^ overall opinion and perceived quality scores ± SD (N = 186) for all study products when presented as conventionally produced (*Conv*), 3D printed (*3DP*), and 3D printed with product benefit information (*3DP + Info*), and mean attitude towards 3D printing ± SD before *3DP* and after *3DP + Info* between and within stratified groups.

Stratified Groups (n)	Overall Opinion			Perceived Quality			Attitude towards 3D Printing	
	Conv	3DP	3DP + Info	Conv	3DP	3DP + Info	Before	After
Food technology neophobia								
FT neophilic	6.9 ± 1.2 ^a^	7.2 ± 1.3 ^bx^	7.3 ± 1.2 ^bx^	3.4 ± 0.9 ^a^	3.9 ± 0.8 ^bx^	3.9 ± 0.8 ^bx^	5.8 ± 0.9 ^ax^	6.4 ± 0.8 ^bx^
(n = 94)								
Less FT neophilic	6.8 ± 1.3	6.6 ± 1.4 ^y^	6.7 ± 1.4 ^y^	3.2 ± 0.9 ^a^	3.4 ± 0.9 ^by^	3.5 ± 0.8 ^by^	4.6 ± 1.0 ^ay^	5.5 ± 1.0 ^by^
(n = 92)								
Previous knowledge about 3D printing								
Knowledgeable	6.9 ± 1.2	6.9 ± 1.3	7.0 ± 1.4	3.4 ± 0.9 ^a^	3.7 ± 0.9 ^b^	3.7 ± 0.9 ^b^	5.4 ± 1.1 ^a^	6.0 ± 1.0 ^b^
(n = 109)								
Not knowledgeable	6.7 ± 1.4	6.8 ± 1.5	7.0 ± 1.3	3.3 ± 0.9 ^a^	3.7 ± 0.9 ^b^	3.6 ± 0.8 ^b^	5.0 ± 1.1 ^a^	5.9 ± 1.1 ^b^
(n = 77)								

^1^ Mean scores with different superscript letters (a, b) in the same row are significantly different within stratified groups; mean scores with different superscript letters (x, y) in the same column are significantly different between stratified groups (*p* ≤ 0.05). ^2^ Overall liking evaluated on 9-point hedonic scales anchored from 1 = “dislike very much” and 9 = “like very much”; agreement of a high quality product evaluated on 5-point Likert scales anchored from 1 = “strongly disagree” and 5 = “strongly agree”; attitude towards 3D printing evaluated on 7-point semantic differential scales anchored from 1 = “negative” and 7 = “positive”.

**Table 5:** Participant comments to support paired preference choice of 3D printed or conventional products ^1^.

	Frequency of Mention (%) ^2^	
	Chocolate Swirl (n = 66)	Potato Smiles^®^ (n = 54)
Preferred 3D printed		
Sensory profile; Good/better texture, taste and flavor, appearance	59	31
Products seem same/similar; No preference	30	15
Support new technology, Interesting, Novel	20	13
Perceived benefits; Creative, custom, appealing design, Cost effective, More efficient production	18	6
Preferred conventional		
Sensory profile; Good/better taste and flavor, texture, aroma	12	43
Not opposed to 3D printed food; Recognize benefits of 3DFP, may become interested in the future		17
Products seem same/similar	11	13
Conventional product is more natural/healthier		9
Lack of knowledge about 3DFP	9	
Lack of visual appeal/ 3D design is not cool enough	5	

^1^ Categories mentioned by at least 5% of participants were included for content analysis. ^2^ Each participant could enter multiple responses therefore frequency percentages add up to more than 100%.

## Data Availability

The data presented in this study are available on request from the corresponding author. The data are not publicly available due to ethical reasons.

## Supplementary Materials

The following supporting information can be downloaded at: https://www.mdpi.com/article/10.3390/foods11060809/s1, Table S1: Product-specific benefit information about 3DFP presented to participants in each sensory panel.

## References

[1] Mantihal S., Kobun R., Lee B.B. (2020). 3D food printing of as the new way of preparing food: A review. Int. J. Gastron. Food Sci..

[2] Sun J., Peng Z., Zhou W., Fuh J.Y.H., Hong G.S., Chiu A. (2015). A Review on 3D Printing for Customized Food Fabrication. Procedia Manuf..

[3] Burke-Shyne S., Gallegos D., Williams T. (2021). 3D food printing: Nutrition opportunities and challenges. Br. Food J..

[4] Lipton J.I., Cutler M., Nigl F., Cohen D., Lipson H. (2015). Additive manufacturing for the food industry. Trends Food Sci. Technol..

[5] Varvara R.A., Szabo K., Vodnar D.C. (2021). 3D food printing: Principles of obtaining digitally-designed nourishment. Nutrients.

[6] Dick A., Bhandari B., Dong X., Prakash S. (2020). Feasibility study of hydrocolloid incorporated 3D printed pork as dysphagia food. Food Hydrocoll..

[7] Kouzani A.Z., Adams S., Whyte D.J., Oliver R., Hemsley B., Palmer S., Balandin S. (2017). 3D Printing of Food for People with Swallowing Difficulties. KnE Eng..

[8] Jagadiswaran B., Alagarasan V., Palanivelu P., Theagarajan R., Moses J.A., Anandharamakrishnan C. (2021). Valorization of food industry waste and by-products using 3D printing: A study on the development of value-added functional cookies. Futur. Foods.

[9] Ramachandraiah K. (2021). Potential development of sustainable 3d-printed meat analogues: A review. Sustainability.

[10] Lupton D. (2017). ‘Download to delicious’: Promissory themes and sociotechnical imaginaries in coverage of 3D printed food in online news sources. Futures.

[11] Lyu J., Hahn K., Sadachar A. (2018). Understanding millennial consumer’s adoption of 3D printed fashion products by exploring personal values and innovativeness. Fash. Text..

[12] Brunner T.A., Delley M., Denkel C. (2018). Consumers’ attitudes and change of attitude toward 3D-printed food. Food Qual. Prefer..

[13] Manstan T., McSweeney M.B. (2020). Consumers’ attitudes towards and acceptance of 3D printed foods in comparison with conventional food products. Int. J. Food Sci. Technol..

[14] Lupton D., Turner B. (2018). “I can’t get past the fact that it is printed”: Consumer attitudes to 3D printed food. Food Cult. Soc..

[15] Caulier S., Doets E., Noort M. (2020). An exploratory consumer study of 3D printed food perception in a real-life military setting. Food Qual. Prefer..

[16] Mantihal S., Prakash S., Bhandari B. (2019). Texture-modified 3D printed dark chocolate: Sensory evaluation and consumer perception study. J. Texture Stud..

[17] De Andrade Silva A.R., Bioto A.S., Efraim P., de Castilho Queiroz G. (2017). Impact of sustainability labeling in the perception of sensory quality and purchase intention of chocolate consumers. J. Clean. Prod..

[18] Kongstad S., Giacalone D. (2020). Consumer perception of salt-reduced potato chips: Sensory strategies, effect of labeling and individual health orientation. Food Qual. Prefer..

[19] Liem D.G., Bolhuis D.P., Hu X., Keast R.S.J. (2016). Short communication: Influence of labeling on Australian and Chinese consumers’ liking of milk with short (pasteurized) and long (UHT) shelf life. J. Dairy Sci..

[20] Manstan T., Chandler S.L., McSweeney M.B. (2021). Consumers’ attitudes towards 3D printed foods after a positive experience: An exploratory study. J. Sens. Stud..

[21] Rolland N.C.M., Markus C.R., Post M.J. (2020). The effect of information content on acceptance of cultured meat in a tasting context. PLoS ONE.

[22] Kuang L., Burgess B., Cuite C.L., Tepper B.J., Hallman W.K. (2020). Sensory acceptability and willingness to buy foods presented as having benefits achieved through the use of nanotechnology. Food Qual. Prefer..

[23] Meier-Dinkel L., Trautmann J., Frieden L., Tholen E., Knorr C., Sharifi A.R., Bücking M., Wicke M., Mörlein D. (2013). Consumer perception of boar meat as affected by labelling information, malodorous compounds and sensitivity to androstenone. Meat Sci..

[24] Demartini E., Gaviglio A., La Sala P., Fiore M. (2019). Impact of information and Food Technology Neophobia in consumers’ acceptance of shelf-life extension in packaged fresh fish fillets. Sustain. Prod. Consum..

[25] Giordano S., Clodoveo M.L., Gennaro B., Corbo F. (2018). Factors determining neophobia and neophilia with regard to new technologies applied to the food sector: A systematic review. Int. J. Gastron. Food Sci..

[26] Lupton D., Turner B. (2018). Food of the Future? Consumer Responses to the Idea of 3D-Printed Meat and Insect-Based Foods. Food Foodways.

[27] Henson S., Annou M., Cranfield J., Ryks J. (2008). Understanding consumer attitudes toward food technologies in Canada. Risk Anal..

[28] Vidigal M.C.T.R., Minim V.P.R., Simiqueli A.A., Souza P.H.P., Balbino D.F., Minim L.A. (2015). Food technology neophobia and consumer attitudes toward foods produced by new and conventional technologies: A case study in Brazil. LWT.

[29] Pliner P., Hobden K. (1992). Development of a Scale to Measure the Trait of Food Neophobia in Humans. Appetite.

[30] Cox D.N., Evans G. (2008). Construction and validation of a psychometric scale to measure consumers’ fears of novel food technologies: The food technology neophobia scale. Food Qual. Prefer..

[31] Zhu X., Xie X. (2015). Effects of knowledge on attitude formation and change toward genetically modified foods. Risk Anal..

[32] Mashable What Is 3D Printing and How Does It Work?|Mashable Explains [Video File]. https://www.youtube.com/watch?v=Vx0Z6LplaMU.

[33] Candel M.J.J.M. (2001). Consumer’s convenience orientation towards meal preparation: Conceptualization and measurement. Appetite.

[34] Steptoe A., Pollard T.M., Wardle J. (1995). Development of a Measure of the Motives Underlying the Selection of Food: The Food Choice Questionnaire. Appetite.

[35] Teo T. (2013). An initial development and validation of a Digital Natives Assessment Scale (DNAS). Comput. Educ..

[36] Erlingsson C., Brysiewicz P. (2017). A hands-on guide to doing content analysis. Afr. J. Emerg. Med..

[37] Barylko-Pikielna N., Matuszewska I., Jeruszka M., Kozlowska K., Brzozowska A., Roszkowski W. (2004). Discriminability and appropriateness of category scaling versus ranking methods to study sensory preferences in elderly. Food Qual. Prefer..

[38] Muthurajan M., Veeramani A., Rahul T., Gupta R.K., Anukiruthika T., Moses J.A., Anandharamakrishnan C. (2021). Valorization of Food Industry Waste Streams Using 3D Food Printing: A Study on Noodles Prepared from Potato Peel Waste. Food Bioprocess Technol..

[39] Da Cunha D.T., Antunes A.E.C., Da Rocha J.G., Dutra T.G., Manfrinato C.V., Oliveira J.M., Rostagno M.A. (2019). Differences between organic and conventional leafy green vegetables perceived by university students: Vegetables attributes or attitudinal aspects?. Br. Food J..

[40] Sleboda P., Lagerkvist C.J. (2022). Tailored communication changes consumers’ attitudes and product preferences for genetically modified food. Food Qual. Prefer..

[41] Su C.H., Tsai C.H., Chen M.H., Lv W.Q. (2019). U.S. sustainable food market generation Z consumer segments. Sustainability.

[42] Galati A., Tulone A., Moavero P., Crescimanno M. (2019). Consumer interest in information regarding novel food technologies in Italy: The case of irradiated foods. Food Res. Int..

[43] Beghin J.C., Gustafson C.R. (2021). Consumer valuation of and attitudes towards novel foods produced with new plant engineering techniques: A review. Sustainability.

[44] Hellwig C., Gmoser R., Lundin M., Taherzadeh M.J., Rousta K. (2020). Fungi Burger from Stale Bread? A Case Study on Perceptions of a Novel Protein-Rich Food Product Made from an Edible Fungus. Foods.

[45] Vanhonacker F., Lengard V., Hersleth M., Verbeke W. (2010). Profiling European traditional food consumers. Br. Food J..

[46] Lupton D., Turner B. (2017). ‘Both Fascinating and Disturbing’: Consumer Responses to 3D Food Printing and Implications for Food Activism.

[47] Siegrist M., Hartmann C. (2020). Consumer acceptance of novel food technologies. Nat. Food.

[48] Lin W., Ortega D.L., Caputo V., Lusk J.L. (2019). Personality traits and consumer acceptance of controversial food technology: A cross-country investigation of genetically modified animal products. Food Qual. Prefer..

[49] Modlinska K., Adamczyk D., Goncikowska K., Maison D., Pisula W. (2020). The effect of labelling and visual properties on the acceptance of foods containing insects. Nutrients.

[50] Stone H., FitzGibbon L., Millan E., Murayama K. (2022). Curious to eat insects? Curiosity as a Key Predictor of Willingness to try novel food. Appetite.

[51] Chen Q., Anders S., An H. (2013). Measuring consumer resistance to a new food technology: A choice experiment in meat packaging. Food Qual. Prefer..

