# Supplementary material for: Effect of Labelling and Information on Consumer Perception of Foods Presented as 3D Printed
Source: Foods. 2022 Mar 11;11(6):809. doi: 10.3390/foods11060809 (PMC8953996; doi:10.3390/foods11060809)
Supplement: Supplementary file 1 [file foods-11-00809-s001.zip › foods-1629682-supplementary.pdf]

Supplementary Table

Table S1. Product-specific benefit information about 3DFP presented to participants in each sensory panel.

**Chocolate swirl panel**
**Chocolate for art and creativity.** 3D printers can easily and efficiently create interesting, novel, and complex designs that would be difficult and time-consuming to replicate by hand. These complex designs are highly customizable through digital design software, providing an opportunity for anyone to create their own designs. This aspect of 3D printing is very appealing to chocolatiers and confectioners who are constantly looking to create innovative chocolates for their consumers, but also for any user who desires to create their own unique and personalized chocolates.
**Gummy candy carrot panel**
**Gummy candy for nutrient delivery, and creating novel and complex designs.** 3D printed foods can potentially create personalized foods containing the correct percentage of nutrients for a particular age or gender. It is important for individuals of all ages to meet their daily nutritional needs, but especially for children in order to have proper growth and development. A 3D printed gummy candy can be formulated to meet the nutritional needs - in terms of energy, vitamins and minerals - of children within a certain age range and at the same time create attractive and complex designs that would peak their curiosity to consume the food.
**Potato Smiles® panel**
**Mashed potatoes for attractive shaping of soft foods.** 3D food printing can be beneficial for the elderly or individuals that suffer from dysphagia; a medical condition in which people have difficulty chewing and swallowing food. Their meals usually consist of soups, liquids, or unappealing pureed globs of food. With a 3D printer, pureed meats and vegetables can be shaped into attractive designs that resemble the original food the material was pureed from. These printed foods could increase appetite, improve nutritional intake and quality of life for those suffering from dysphagia.
